# Improving the Efficiency of the Intravenous Medicine Preparation Pathway With an Intravenous Workflow Software Solution in Full-Capacity Pharmacy Units at Watford General Hospital: Observational Study and Economic Analysis

**DOI:** 10.2196/85408

**Published:** 2026-02-18

**Authors:** Bethany Umpleby, Nick Hex, Teresa Martins, Husanain Soori

**Affiliations:** 1 York Health Economics Consortium York United Kingdom; 2 West Hertfordshire Hospitals NHS Trust Watford United Kingdom

**Keywords:** BD Cato Pharmacy, intravenous, compounding software, anticancer therapy, efficiency, automation

## Abstract

**Background:**

The existing intravenous systemic anticancer therapy (SACT) pathway in pharmacies is operationally inefficient. Manual, paper-based workflows render the system prone to human error, and the need for time-consuming manual verification diverts pharmacy staff time. The introduction of an automated workflow solution for the intravenous SACT pathway could optimize treatment timeliness and improve oncological outcomes for patients, aligning with the National Health Service Long Term Plan for improved cancer care.

**Objective:**

This observational analysis aimed to assess the change in time, cost, and errors following the implementation of the Becton Dickinson (BD) Cato Pharmacy system in an aseptic unit producing intravenous SACT at Watford General Hospital.

**Methods:**

Data on compounding process times were collected manually by pharmacy staff before and after the implementation of the intravenous compounding software (BD Cato). The data were analyzed to estimate annual time savings, opportunity cost savings, and error reduction.

**Results:**

The intravenous compounding software produced a time saving of 18 (SD 9) minutes per drug, equating to 1034 hours saved per year (1034/2591, 39.9% reduction). If this time were repurposed to producing more intravenous SACT, Watford General Hospital could increase production by 66% (2298/3482) annually (2298 additional intravenous SACT). This represents an average cost saving of £11.29 (£1=US $1.273) per drug, equating to an annual opportunity cost saving of £39,246. The intravenous compounding software also decreased observed errors by 86% (43/50), a reduction of 43 errors over 2 months (approximately 258 fewer errors annually). Staff also preferred the intravenous compounding software to the manual system.

**Conclusions:**

Implementing intravenous compounding software can save time, reduce costs, and lower errors in intravenous SACT preparation. This could improve timely treatment access for patients with cancer.

## Introduction

The National Health Service (NHS) Long Term Plan includes targets for the NHS in the United Kingdom, including better care for cancer [1]. NHS England made a case for transforming pharmacy aseptic services, including reducing medical waste to reduce NHS costs [2], and reducing medication errors [3]. As medication errors are approximately 5 times more likely to occur for intravenous medications [4] and can cause significant patient harm [5], there is increasing pressure on aseptic units to reduce this error risk [2,6].

In 2022, the British Oncology Pharmacy Association found that 91% of respondents experienced same-day treatment delays due to delays within their pharmacy’s service [7]. Delays in preparation within the pharmacy aseptic unit accounted for 69% of these pharmacy-related delays to intravenous systemic anticancer therapy (SACT) administration. Similarly, 76% of respondents had their treatment rescheduled at least once in 6 months [7]. Two main causes resulting in the rescheduling of treatment were known staffing issues (33%) and aseptic unit capacity (35%). This indicates that the current pathway for the preparation of intravenous medicines in full-capacity aseptic units is struggling to manage these demands.

The traditional pathway for the production of intravenous SACT involves a lengthy process, characterized by a manual paper-based workflow, which is slow, susceptible to errors, and lacks traceability. There is also a heavy reliance on manual verification and supervision to ensure a quality-assured process, which requires substantial pharmacy staff time. As the number of patients requiring first-line chemotherapy is estimated to increase each year [8], an alternative automated solution is required to improve efficiency, increase capacity, reduce costs, and reduce the risk of errors [2,9]. Becton Dickinson (BD) has developed a medication workflow software solution, BD Cato, which offers a potential solution to help organizations address these challenges.

A study from 2021 found that using the BD Cato software in East Tallinn Central Hospital, Estonia, reduced compounding time by 35% and doubled medication production capabilities over the course of the study [10]. BD Cato Pharmacy has also been found to detect errors in 7.89% of prepared cancer drugs, which would not have otherwise been detected using traditional compounding methods [11]. Therefore, the BD Cato Pharmacy software has the potential to save time (and potentially save costs) and to reduce errors, thus helping to meet policy targets.

The aim of this economic analysis was to assess the observed change in time, cost, and error rates associated with the implementation of the BD Cato Pharmacy system within an aseptic unit producing intravenous SACT at Watford General Hospital (WGH), England.

## Methods

### The Intravenous Compounding Software

BD Cato is a medication workflow solution software that standardizes prescription, compounding, and administration processes along the medication pathway.

BD Cato Pharmacy is the module within this workflow related to the compounding process and provides functionality for the gravimetric and volumetric preparation of prescribed intravenous medication, such as chemotherapy, parenteral nutrition, pain medication, and antibiotics. It supports barcode scanning of vials and other components for electronic verification and documentation, automated calculations, step-by-step on-screen workflow guidance, gravimetric preparation using an electronic scale, image capture of preparation steps via a camera, hard stops when deviations have been detected, inventory and remainder management functionality, and production planning and report generation.

**Figure 1:**
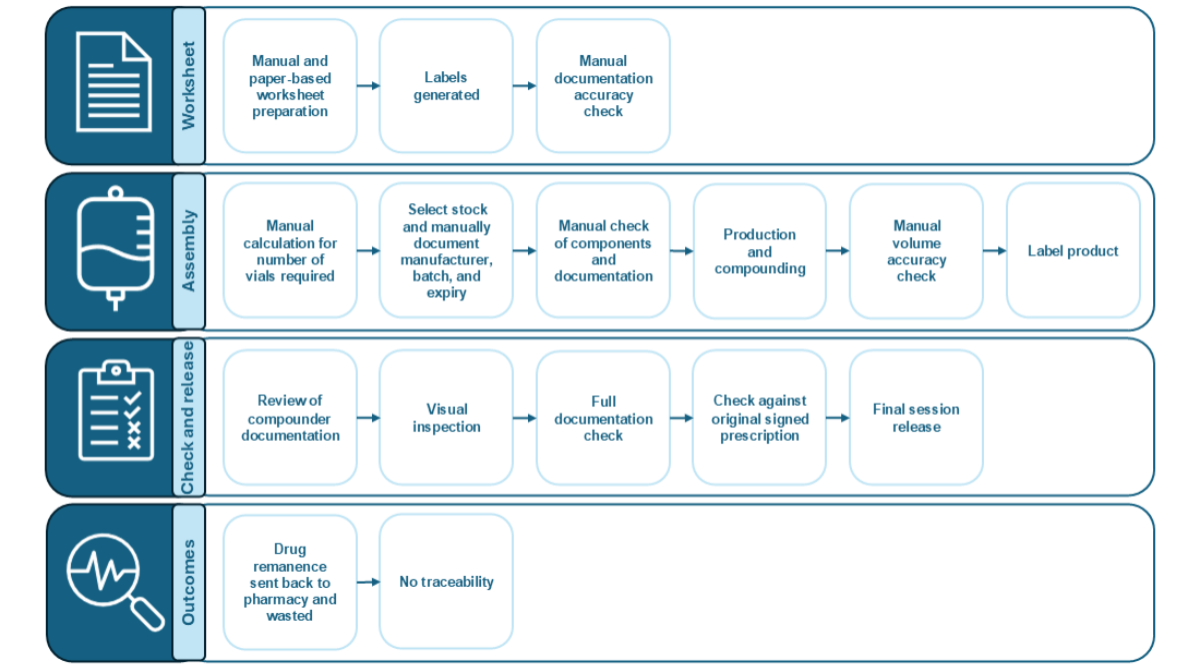
Current pathway for the preparation of intravenous medications for cancer.

**Figure 2:**
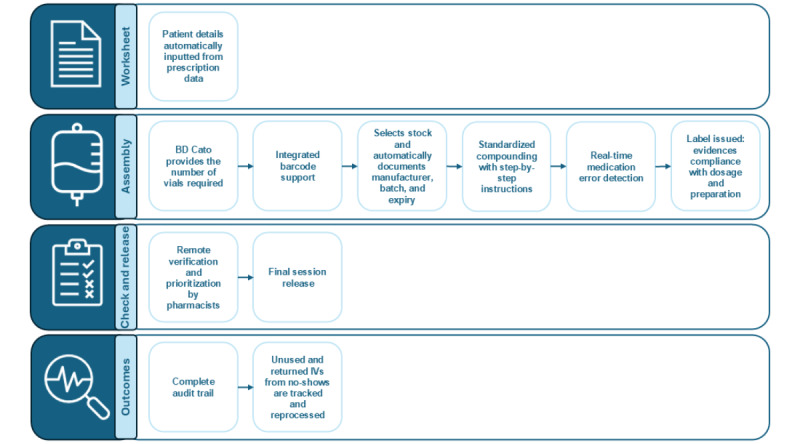
The Becton Dickinson (BD) Cato Pharmacy pathway for the preparation of intravenous (IV) systemic anticancer therapy for cancer in this study.

Figure 1 demonstrates the pathway for the preparation of intravenous SACT without the use of BD Cato. Figure 2 demonstrates the pathway for the preparation of intravenous SACT with the use of BD Cato.

### Study Design

This was a single-center before-and-after observational study. Data collection was carried out in the pharmacy department at WGH, which had decided to adopt the BD Cato system, and was interested in understanding the impact on time, costs, and error rates. The period for data collection before the introduction of the intravenous compounding software was approximately 10 weeks, between March 2021 and May 2021. The period for data collection after the introduction of the intravenous compounding software was approximately 8 weeks, between December 2023 and January 2024.

A data collection sheet was produced in collaboration with a qualified pharmacist at WGH to record the metrics shown in Textbox 1.

Pharmacy staff at WGH were asked to manually complete data collection sheets for every intravenous SACT that was compounded within the period before and after the implementation of the intravenous compounding software. Before and after the implementation of the intravenous compounding software, manual data collection methods were used, whereby pharmacy staff were asked to record the time it took them to complete different aspects of the manufacturing process on a paper form. These were collated and analyzed by the York Health Economics Consortium.

The time to compound and reconstitute intravenous SACT and the number and types of errors before the study were unknown. This made sample size calculation difficult to determine. A period of 8 to 10 weeks was chosen to allow sufficient numbers of intravenous SACT compounding data to be collected and to be able to accurately measure the time taken for the compounding of different classes of intravenous SACT. It also provided time to record any errors that occurred during the data collection periods.

**Summary of primary metrics and parameters captured during the data collection process.**

**Metrics recorded in the data collection sheet**

- The intravenous systemic anticancer therapy drug name being compounded
- The time taken for each task
  Worksheet completion
  Worksheet checking
  Assembly
  Assembly checking
  Compounding
  Labeling
  Check and release
- Any identified errors
  Wrong stability entered
  Wrong diluent or fluid
  Wrong volume drawn in compounding
  Missing assembly item
  Wrong batch number (assembly)
  Wrong protocol
  Wrong vial size chosen
  Wrong volume entered
  Wrong dose inputted
  Wrong dosage form
  Wrong or no ward
  Missing or wrong infusion time
  Other (please state)

Data from the data collection sheets were manually input into Google Sheets to clean and store the data and perform basic data analysis. Once data collection was complete, the data were exported, and analysis was performed in Microsoft Excel.

The primary outcome of the study was the impact of the intravenous compounding software on the time taken to compound intravenous SACT. The secondary outcomes were the impact of the intravenous compounding software on direct costs and the number of errors made during the compounding of intravenous SACT.

In addition to the quantitative analysis, 2 surveys were distributed among pharmacy staff members: 1 before the implementation of the intravenous compounding software and 1 after the implementation of the intravenous compounding software (18 months apart). The objective was to assess staff perception of the BD Cato workflow compared to the previous manual process with regard to safety, accuracy, efficiency, visibility of production status, work satisfaction, and overall preference. It was also used to evaluate staff satisfaction with the introduction of the new system.

### Ethical Considerations

This study did not require ethics approval as the analysis was based on a system to automate pharmacy processes and did not involve human participants. Deidentified data on pharmacy processes were retrieved from WGH via staff surveys. West Hertfordshire Teaching Hospitals NHS Trust provided access and permission to reuse the dataset for this study.

## Results

### Overview

Preimplementation and postimplementation data for 12 drug types were available from the intravenous compounding software. These drugs are presented in Table 1. Within the preimplementation data, there were an additional 3 drug types that were not present in the postimplementation data: brentuximab, cisplatin, and cytarabine. Within the postimplementation data, there were an additional 5 drug types that were not present in the preimplementation data: bleomycin, carfilzomib, tebentafusp, tocilizumab, and vinblastine. The preimplementation data comprised 408 total preparations, and the postimplementation data comprised 345 total preparations. Average time savings per drug, average time savings per drug per year, and average staff cost savings per drug were calculated. The activities involved in compounding were grouped into production, verification, and check and release. Production consisted of worksheet creation, assembly, compounding, and labeling tasks. Verification consisted of worksheet checking and assembly checking tasks. Check and release consisted of an accuracy and final release check to ensure the medication has been prepared correctly and is suitable for administration.

**Table 1:** Average time saved.

Drug		Production, n	Verification, n	Check and release, n	Total, n
**Average time saved per drug (min), mean (SD)**					
	All: nondrug-specific	12 (0.24)	5 (0.16)	1 (0.15)	18 (0.55)
	Azacitidine	7 (0.42)	5 (0.21)	0 (0.32)	12 (0.94)
	Bendamustine	16 (1.23)	6 (0.33)	–1 (0.83)	21 (2.39)
	Bortezomib	14 (0.79)	5 (0.31)	3 (0.51)	21 (1.61)
	Cyclophosphamide	4 (0.65)	6 (0.37)	0 (0.42)	9 (1.45)
	Dacarbazine	6 (1.44)	6 (0.79)	2 (0.97)	14 (3.20)
	Doxorubicin	7 (0.62)	4 (0.26)	2 (0.35)	13 (1.22)
	Isatuximab	18 (2.37)	6 (1.04)	2 (1.21)	26 (4.62)
	Obinutuzumab	9 (0.91)	7 (0.86)	–2 (0.73)	14 (2.49)
	Polatuzumab	32 (2.78)	4 (1.22)	1 (1.32)	37 (5.32)
	Rituximab (truxima)	10 (0.60)	4 (0.21)	1 (0.39)	14 (1.20)
	Vincristine	8 (0.91)	8 (0.77)	1 (0.51)	17 (2.19)
	Vincristine P	28 (1.38)	7 (0.54)	2 (1.10)	37 (3.01)
**Average time saved per year (h)^a^**					
	All: nondrug-specific	696	293	45	1034
	Azacitidine	69	48	4	121
	Bendamustine	67	25	–3	89
	Bortezomib	123	40	21	184
	Cyclophosphamide	19	29	–2	45
	Dacarbazine	8	9	3	20
	Doxorubicin	28	15	7	49
	Isatuximab	31	11	4	46
	Obinutuzumab	22	17	–5	33
	Polatuzumab	68	8	2	78
	Rituximab (truxima)	68	24	5	97
	Vincristine	11	11	1	23
	Vincristine P	49	12	3	65

^a^The average time multiplied by the amount of activity (SD could not be calculated).

### Time Outcome Results

Time outcomes were examined by calculating the difference between the time taken to complete each activity type in the drug compounding process before and after the implementation of the intravenous compounding software.

Table 1 presents the average time savings per drug compounded in minutes after the introduction of the intravenous compounding software. It presents a nondrug-specific weighted average time saving for each activity group, which included drugs only in the preimplementation phase, drugs only in the postimplementation phase, and drugs included in both data collection phases. It also presents the average time savings for each of the 12 drugs that had both preimplementation and postimplementation data.

Table 1 also presents the average time savings per year in hours. These values were calculated by multiplying the average time saved per drug by the number of each drug compounded per year. This is split into an average nondrug-specific time saving per year and individual time savings per drug per year.

Table 1 shows an average nondrug-specific time saving of 18 (SD 0.55) minutes per drug when using the intravenous compounding software. Time savings were observed for production, verification, and check and release tasks for all drugs, except for check and release for bendamustine and obinutuzumab. However, this did not influence the total average time saved, as all drugs demonstrated a total average time saving. The greatest time saving was observed for production (worksheet, assembly, compounding, and labeling tasks) and for polatuzumab and vincristine P.

Table 1 also shows an average nondrug-specific time saving of 1034 hours per year when using the intravenous compounding software. This represents a 39.9% (1034/2591) reduction in compounding time. This was calculated by multiplying the weighted average time saved per drug (hours) by the 3476 drugs compounded annually by WGH. The greatest time saving for an individual drug was for bortezomib. The individual drug-specific time savings per year were calculated using the number of each drug produced annually at WGH.

Table 2 presents the average time savings per drug per day.

**Table 2:** Average time saved per drug per day (minutes).

Day	Production, mean (SD)	Verification, mean (SD)	Check and release, mean (SD)	Total, mean (SD)
Monday	16 (0.60)	6 (0.36)	1 (0.33)	24 (1.28)
Tuesday	13 (0.57)	5 (0.20)	0 (0.34)	19 (1.10)
Wednesday	8 (0.57)	7 (0.54)	2 (0.35)	17 (1.45)
Thursday	6 (0.47)	5 (0.18)	0 (0.26)	10 (0.92)
Friday	9 (0.56)	5 (0.21)	1 (0.35)	15 (1.13)

Table 2 shows that when using the intravenous compounding software, there were observed time savings on every day of the week. These time savings could not be weighted as data on the number of drugs compounded each day were not available.

### Opportunity Cost Savings

Opportunity cost savings were calculated by multiplying the average time saved by the average staff cost per hour for each task. The average staff cost per hour was based on the staff bands responsible for each task, taken from the Personal Social Services Research Unit [12]. Correspondence with WGH established that production tasks were carried out by staff in bands 3, 4, and 5; verification tasks by staff in bands 5, 6, and 7; and check and release tasks by staff in bands 7 and 8.

The average cost saving per drug and the average cost saving per year are presented in Table 3. This is split into a weighted average nondrug-specific cost saving, which included drugs only in the preimplementation phase, drugs only included in the postimplementation phase, and drugs included in both data collection phases. It also presents individual cost savings per drug for drugs that had both preimplementation and postimplementation data.

**Table 3:** Average cost saving (£1=US $1.273).

Drug		Production (£)	Verification (£)	Check and release (£)	Total (£)
**Average cost saving per drug^a^**					
	All: nondrug-specific	5.98	4.22	1.10	11.29
	Azacitidine	3.50	4.11	0.59	8.20
	Bendamustine	8.10	4.99	–1.10	11.99
	Bortezomib	7.14	3.85	3.53	14.52
	Cyclophosphamide	1.87	4.79	–0.57	6.09
	Dacarbazine	2.96	5.33	2.51	10.80
	Doxorubicin	3.72	3.45	2.50	9.67
	Isatuximab	8.71	5.28	3.28	17.27
	Obinutuzumab	4.40	5.71	–3.03	7.08
	Polatuzumab	16.04	3.14	1.62	20.80
	Rituximab (truxima)	4.94	2.93	0.96	8.83
	Vincristine	4.12	6.59	1.34	12.05
	Vincristine P	14.13	5.78	2.72	22.62
**Average cost saving per year^a^**					
	All: nondrug-specific	20,773	14,661	3811	39,246
	Azacitidine	2052	2412	349	4813
	Bendamustine	2009	1238	–273	2973
	Bortezomib	3661	1977	1811	7449
	Cyclophosphamide	561	1436	–171	1826
	Dacarbazine	249	448	211	907
	Doxorubicin	821	763	553	2137
	Isatuximab	923	559	348	1831
	Obinutuzumab	643	833	–442	1033
	Polatuzumab	2021	396	205	2621
	Rituximab (truxima)	2041	1208	398	3647
	Vincristine	329	527	107	964
	Vincristine P	1469	601	283	2353

^a^The average time multiplied by the amount of activity (SD could not be calculated).

Table 3 shows a weighted average nondrug-specific total cost saving of £11.29 (£1=US $1.273) per drug. Table 3 also shows a weighted average nondrug-specific total cost saving of £39,246 per year. The use of the intravenous compounding software was observed as cost saving for all production tasks, all verification tasks, and most check and release tasks, excluding bendamustine, cyclophosphamide, and obinutuzumab, which were cost incurring. Nevertheless, this did not have an impact on the total cost savings of these drugs, as all drugs resulted in a total opportunity cost saving. The greatest opportunity cost savings per year were observed for production tasks and for bortezomib.

### Error Outcome Results

The reduction in the number of observed errors from the use of the intravenous compounding software was calculated by finding the difference between the number of errors reported before and after its introduction. These results are presented in Table 4. While the intravenous compounding software anticipates potential errors to proactively implement mitigations and prevent process inefficiencies, this analysis focused solely on observed errors that directly resulted in process inefficiencies.

**Table 4:** Number of errors saved.

Error summary	Before the use of BD^a^ Cato Pharmacy software (n=50), n (%)	After the use of BD Cato Pharmacy software (n=7), n (%)	Difference (n=43), n (%)
Wrong stability entered	0 (0)	0 (0)	0 (0)
Wrong diluent or fluid	0 (0)	0 (0)	0 (0)
Wrong volume drawn in compounding	3 (6)	0 (0)	3 (7)
Missing assembly item	27 (54)	1 (14)	26 (60)
Wrong batch number (assembly)	8 (16)	1 (14)	7 (16)
Wrong protocol	1 (2)	0 (0)	1 (2)
Wrong vial size chosen	5 (10)	1 (14)	4 (9)
Wrong volume entered	0 (0)	0 (0)	0 (0)
Wrong dose inputted	0 (0)	0 (0)	0 (0)
Wrong dosage form	0 (0)	0 (0)	0 (0)
Wrong or no ward	1 (2)	0 (0)	1 (2)
Missing or wrong infusion time	0 (0)	0 (0)	0 (0)
Other	5 (10)	4 (57)	1 (2)

^a^BD: Becton Dickinson.

Table 4 shows that the intravenous compounding software decreased the risk of observed errors by 86% (43/50). There was a reduction of 43 observed errors over a 2-month period, which equates to reducing approximately 258 observed errors per year. The greatest reduction in errors was for missing assembly items. The intravenous compounding software reduced errors through barcode scanning, automatic documentation, vial size suggestion, and guided workflows, preventing staff from proceeding to the next step until the current step was correctly completed. There were 9 reported cases of the intravenous compounding software losing signal, which required staff to log out and log back in to continue preparation. While these were not reported as errors, as they did not result in loss of data or require a workflow step to be repeated, they resulted in an increase in time taken to prepare the drug, which is accounted for in the time and opportunity cost saving analysis.

### Staff Survey Results

The preimplementation survey had a response rate of 93% (13/14), and the postimplementation survey had a response rate of 100% (8/8). A total of 88% (7/8) of postimplementation respondents preferred to compound a product using the intravenous compounding software and perceived it as being safer, more accurate, and more efficient compared to a manual workflow. In total, 100% (8/8) of respondents believed that the intravenous compounding software facilitates the tracking of sterile products during the compounding process. Regarding work satisfaction, none of the respondents found work dissatisfying using the intravenous compounding software, compared to 8% (1/13) of respondents who found work dissatisfying using the previous manual workflow.

Before receiving training on the use of the intravenous compounding software, preimplementation staff were asked an open-ended question about what they considered to be its potential advantages. The most common response was improvement in process efficiency, with 1 respondent stating that freeing up time would allow more focus on clinical roles related to oncology. The second most common responses were reduction of errors and improved accuracy. Traceability, removing the need for paperwork, waste reduction, and cost savings were also perceived as advantages. One respondent also perceived that the new system would upskill staff in the use of technology.

Staff in the preimplementation survey were also asked an open-ended question on what they would consider the likely challenges or disadvantages of introducing the intravenous compounding software. The most common response was related to overcoming resistance to change and having to adopt new ways of working due to familiarity with old processes and procedures. The second most common response was training requirements, with some respondents acknowledging that it can be a challenge for staff that are lacking in IT skills to adapt to new technology. Other perceived challenges were the need for a downtime plan and new types of errors that could be introduced.

## Discussion

### Principal Findings

In this observational study, the use of an intravenous compounding software reduced time, costs, and errors during the production of intravenous SACT at WGH. It was estimated that the use of the intravenous compounding software would save 1034 hours of pharmacy staff time per year. If this time were all repurposed toward the production of intravenous SACT, an additional 2298 drugs could be compounded at WGH each year. This is an increase of approximately 66.11% (2298/3476) from the number of intravenous SACT currently being produced by WGH per year (n=3476). If half of the time saved (517 hours) was repurposed toward the production of intravenous SACT, an additional 1149 drugs could be compounded at WGH each year. It was observed that the use of an intravenous compounding software reduced the time to produce, verify, and check and release intravenous SACT by 39.9% (1034/2591). These results are in line with previous research that also found that the intravenous compounding software produced a 35% reduction in compounding time [10].

On the basis of the time saved and the average staff cost to the NHS, the observed time saved using the intravenous compounding software can be valued at £39,246 per year. This figure can be viewed as an opportunity cost saving, as the time saved could be reinvested into producing more intravenous SACT without incurring additional labor costs. The opportunity cost of an intervention is what is foregone as a consequence of adopting a new intervention [13].

There was some heterogeneity in the observed time savings between different drugs, partly driven by the number of steps in the manual manufacturing process for certain drugs. Bendamustine, in particular, is a complex product to make and typically involves more vials to reconstitute and larger volumes to be measured and then transferred to the infusion bag. As there are more steps in the process, the pharmacist review contains more photos of the steps in the compounding process for the releasing officer to review, and therefore, it would take longer to complete.

The greatest time savings per drug were on a Monday, and the least time savings per drug were on a Thursday. One explanation for this is that different levels of staff may work on different days throughout the week. Another explanation is that the number and types of drugs compounded per day may differ; for example, drugs that are slower to prepare may be compounded on a specific day.

The use of an intravenous compounding software was also shown to decrease the occurrence of errors requiring activity to be redone by 86% (43/50), with the potential to reduce 258 errors per year at WGH. This is in line with previous research that also reported the error-reducing capabilities of BD Cato Pharmacy [11]. It is of interest that there were 0 diluent errors before implementation. This is at odds with a survey carried out by the Institute for Safe Medication Practices, which found that more than half of pharmacists and pharmacist technicians who responded could identify with certainty which drugs, diluents, and volumes were used when verifying compounded sterile preparations [14]. WGH solely used volumetric preparation before implementation, but after implementation, a mix of both volumetric and gravimetric preparations was used, with most products made gravimetrically.

Therefore, the introduction of an intravenous compounding software could contribute toward meeting NHS targets that are currently unmet. For example, the British Oncology Pharmacy Association survey found that one of the main causes of same-day treatment delays was delays within the pharmacy aseptic unit (69%), and one of the main causes of rescheduled treatments was limited aseptic unit capacity (35%) [7]. Therefore, if more intravenous SACT can be compounded more quickly using an intravenous compounding software within aseptic units, same-day treatment delays and rescheduled treatments could be reduced. This could also help to manage the predicted increase in treatments required in the coming years [8].

The implementation of the intravenous compounding software was well received by staff who completed the postimplementation survey. The preimplementation open-ended question on perceived challenges highlights the importance of getting staff on board with the change at an early stage and taking steps to overcome resistance. Sufficient time and resources should also be allocated to training to support the effective use of the system and help overcome the concerns and resistance that staff have.

### Limitations

This analysis has several limitations. Because the data were collected manually, there is an increased risk of human error when completing the data collection sheet, as well as potential inconsistencies in how individuals report different data items.

There are also potential confounding variables that may have influenced the findings of the study. Data were collected at 2 different time points, with preimplementation data collected in 2021 and postimplementation data collected in 2023 to 2024. Factors that may have influenced the data collection process between 2021 and 2023 to 2024 include changes in protocols or procedures, changes in staff (including staff competency), and changes in resources other than the intravenous compounding software. Similarly, data were collected over short periods, which might not capture long-term effects or trends. Longitudinal effects or outcomes might differ from these short-term findings.

A similar limitation applies to the staff survey. Preimplementation survey responses were collected in 2021, while postimplementation survey responses were collected between 2022 and 2024. Due to staff turnover, respondents may differ before and after the implementation of the intravenous compounding software. It is also uncertain whether all respondents had experience with compounding using the previous manual process.

The study was not intended to be a return on investment analysis. The time saved has been quantified in financial terms to provide a context for the benefits of automation. Consequently, the investment required for the installation and maintenance of the system was not taken into consideration when savings were calculated. Similarly, no calculation was made of the investment WGH would have required to achieve comparable productivity gains had it retained the previous manual workflow.

In response to the limitations of this study, future research could address the following areas. The economic impact of BD Cato Pharmacy across other aseptic units within the NHS, and for different types of drugs (eg, intravenous antibiotics or nutrition), should be investigated. This would be beneficial to assess the broader applicability of the results. In addition, future research could investigate the cost impact of managing drug remnants and drug waste using BD Cato Pharmacy’s remnants functionality.

### Conclusions

The aim of this economic analysis was to assess the observed change in time, cost, and error rates associated with the implementation of an intravenous compounding software system within an aseptic unit producing intravenous SACT at WGH. BD Cato Pharmacy has the capability to reduce time, costs, and errors and offers a practical and scalable solution to address key areas within the NHS, such as improving efficiency and increasing capacity. The system’s ability to proactively implement mitigations helps to provide necessary transparency for medication management. The system requires some IT infrastructure to operate, including hardware (barcode scanners, label printers, a printer, keyboards, a PC with Windows 10, scales, and visual documentation hardware) and software (SQL server with 4 central processing unit cores and 16 GB memory random-access memory, Microsoft .NET Framework [version 4.8.0], and DirectX [version 9.0]).

## Abbreviations

BD: Becton Dickinson
NHS: National Health Service
SACT: systemic anticancer therapy
WGH: Watford General Hospital

## Data Availability

All data generated or analyzed during this study are included in this published paper. The datasets generated or analyzed are available from the corresponding author upon reasonable request.
